# Oligonucleotides—A Novel Promising Therapeutic Option for IBD

**DOI:** 10.3389/fphar.2019.00314

**Published:** 2019-04-24

**Authors:** Patrizio Scarozza, Heike Schmitt, Giovanni Monteleone, Markus F. Neurath, Raja Atreya

**Affiliations:** ^1^Department of Systems Medicine, Gastroenterology, University of Tor Vergata, Rome, Italy; ^2^Department of Medicine 1, Friedrich-Alexander-University of Erlangen-Nürnberg, Erlangen, Germany

**Keywords:** Crohn disease, ulcerative colitis, IBD, antisense oligonucleotide (ASO), target therapies

## Abstract

Inflammatory Bowel Diseases (IBD), whose denomination comprehends Crohn's Disease (CD) and Ulcerative Colitis (UC), are intestinal chronic diseases that often require lifelong medical therapy. In the last two decades monoclonal antibodies against the cytokine TNF have become integral parts in the treatment of IBD patients, however there are unwanted side-effects and one third of patients show primary non-response while another subgroup loses response over time. Finding novel drugs which could act as therapies against precise pro-inflammatory molecular targets to avoid unwanted systemic side effects and additionally the process of immunization, represents an important aim for subsequent therapeutic approaches. Oligonucleotide based therapies represent a promising novel concept for the treatment of IBD. The molecular action of oligonucleotides ranges from inhibition of the translational process of mRNA transcripts of pro-inflammatory molecules, to mimicking bacterial DNA which can activate cellular targets for immunomodulation. Alicaforsen, selectively targets ICAM-1 mRNA. ICAM-1 is an adhesion molecule which is upregulated on endothelial cells during IBD, thereby mediating the adhesion and migration of leucocytes from blood to sites of active inflammation. In CD parenteral application of alicaforsen did not show therapeutic efficacy in phase II trials, but it demonstrated an improved efficacy as a topical enema in distal UC. Topical application of alicaforsen might represent a therapeutic perspective for refractory pouchitis as well. SMAD7 is a protein that inhibits the signaling of TGFβ, which is the mainstay of a regulatory counterpart in cellular immune responses. An antisense oligonucleotide against SMAD7 mRNA (mongersen) demonstrated pre-clinical and phase II efficacy in CD, but a phase III clinical trial was stopped due to lack of efficacy. Cobitolimod is a single strand oligonucleotide, which mimics bacterial DNA as its CpG dinucleotide sequences can be recognized by the Toll-like receptor 9 on different immune cells thereby causing induction of different cytokines, for example IL10 and IFNα. Topical application of cobitolimod was studied in UC patients. We will also discuss two other novel oligonucleotides which act on the GATA3 transcription factor (SB012) and on carbohydrate sulfotransferase 15 (STNM01), which could both represent novel promising therapeutic options for the treatment of UC.

## Introduction

Inflammatory bowel diseases (IBD) are chronic inflammatory disorders of the intestine whose phenotypic spectrum mainly comprehends Crohn's disease (CD) and ulcerative colitis (UC). IBD are characterized by a chronic disease course with the alternation of active and quiescent phases, thereby requiring continuous follow up and management by health care systems (Maaser et al., 2019). In the last 20 years, there has been an increase in the prevalence of IBD from a percentage of 0.3–0.5% in North America and similarly in Northern Europe, Western Europe, and Oceania (Kaplan and Ng, 2017). Moreover, a profound rise in incidence was described in Asia and Latin America (Ng et al., 2018).

IBD patients often require treatment with immunomodulatory drugs such as thiopurines, methotrexate; biological drugs, like anti-Tumor Necrosing Factor (anti-TNF), anti-adhesion, or anti-interleukin 12/23p40 (IL12/23p40) antibodies and lately also Janus Kinase Inhibitors (JAK-inhibitors). These different substance classes have become integral parts in the treatment of IBD patients; however there are unwanted, sometimes even severe side effects and over one third of patients show primary non-response while another subgroup of patients loses response over time (Roda et al., 2016). There is therefore the dire need for novel efficacious therapies with a favorable safety profile. Oligonucleotides represent an emerging substance class of targeted therapies with only limited systemic exposure to the drug, giving hope for a novel and rather safe treatment modality in IBD.

The molecular mechanism of action of oligonucleotides ranges from inhibition of the translational process of messenger ribonucleic acid (mRNA) transcripts of pro-inflammatory molecules, to mimicking bacterial deoxyribonucleic acid (DNA) which can activate cellular targets for immunomodulation.

Antisense technology is based on two principal different modes of action: activation of Ribonuclease H (RNase H) or double-strand RNases. The activation of RNase H is mediated by the recognition of the sense-antisense heterodimer, after the binding of the single-strand oligonucleotide to the complementary target mRNA (Crooke, 2004). The heterodimer induces the activation of the RNase H enzyme, which inhibits the expression of the target mRNA (Aboul-Fadl, 2005). The second antisense mechanism is linked to the action of double-strand RNases which are activated by silence RNA (siRNA) molecules that are characterized by a double-strand oligonucleotide formed by a sense and an antisense RNA strand. After entering the cell, the antisense RNA recognizes the target cytoplasmic mRNA, forming a sense-antisense duplex, that is finally degraded by endogenous double-strand RNases (Crooke, 2004).

For the last 20 years, antisense technology has been improved in various fields of medical research. In 1999 the European Agency for the Evaluation of Medical Products (EMEA) approved Fomivirsen, as the first therapeutically used antisense oligonucleotide, developed for the treatment of Cytomegalovirus (CMV) retinitis. In the meantime many more oligonucleotide based substances have been tested in respective trials or already entered clinical practice: Macugen to treat age-related macular degeneration; Mipomersen for homozygous familial hypercholesterolemia; Eteplirsen for Duchenne muscular dystrophy; Defibrotide for severe hepatic veno-occlusive disease; and Nusinersen for the treatment of infants with types 1, 2, and 3 spinal muscular atrophy (Stein and Castanotto, 2017).

This review focuses on pharmacodynamics and clinical perspectives of oligonucleotide based therapeutics so far studied for the treatment of IBD. (Supplementary Material).

## Alicaforsen

Active inflammation in IBD is characterized by overexpression of adhesion molecules on endothelial cells that are centrally involved in mediating the gut homing of leukocytes. Inhibition of migration has become one of the established therapeutic approaches in IBD, with approval of the alpha4beta7 antibody vedolizumab for treatment of both IBD entities and ongoing clinical trials for many more therapeutic targets of the homing process (Sands, 2017; Zundler and Neurath, 2017). Intercellular Cell Adhesion Molecule-1 (ICAM1) is an adhesion molecule derived from the immunoglobulin superfamily acting as a ligand for the integrins expressed by leukocytes and represents one of the novel targets of antisense oligonucleotide (ASO) based therapeutic strategies (Mosli et al., 2014; Sands, 2017).

ICAM1 is overexpressed by endothelial cells during active disease in CD patients and its presence is thus diminished during remission. It was furthermore shown, that in UC patients the expression of ICAM1 in colonic tissue is higher than in respective controls (Jones et al., 1995; Vainer and Nielsen, 2000; Rivera-Nieves et al., 2008).

Preclinical studies have figured out the efficacy of a monoclonal antibody against ICAM1 administered topically in the experimental dextran sulfate sodium (DSS) murine model of colitis, which led to the reduction of mucosal inflammation (Hamamoto et al., 1999). When combined with anti-Vascular Cell Adhesion Molecule-1 (anti-VCAM1) or anti-alpha4 integrin antibodies, the ICAM1 antibody similarly exerted a positive effect on reducing the inflammatory response in a murine model of ileitis (Burns et al., 2001).

Alicaforsen is a 20-base phosphorothioate ASO that hybridizes the mRNA of ICAM1 and induces the hydrolysis of the DNA-RNA complex by an RNAse enzyme (Gewirtz and Sitaraman, 2001). A randomized trial of ISIS 2302, an ASO to ICAM1, was conducted in patients affected by severe rheumatoid arthritis, without evidence of significant efficacy, but with a good safety profile (Maksymowych et al., 2002). A pivotal phase I dose ranging trial conducted on healthy volunteers who intravenously received the ICAM1 ASO, showed good tolerance to the drug, with reproducible plasma levels and limited side effects (dose-related increase in activated partial thromboplastin time, clinical insignificant increases in complement factor C3a) at the time of the peak plasma concentration, without occurrence of associated clinical symptoms (Glover et al., 1997).

A dose ranging pharmacokinetic trial of high-dose alicaforsen randomized 22 patients with active CD to three alicaforsen treatment groups: 250-, 300-, or 350-mg doses, infused intravenously three times a week for 4 weeks. The authors evidenced a possible effectiveness of alicaforsen therapy in CD. Clinical remission was evidenced in 41% of all treated subjects and in 53% of those treated with more than three of the 12 infusions planned (Yacyshyn et al., 2002a).

A randomized trial of ISIS 2302, conducted in twenty CD patients, allocated to receive one of three different doses of this drug (0.5, 1, or 2 mg/kg) or saline placebo, evidenced a therapeutic success of the experimental drug, which was infused intravenously in 13 doses over 26 days (Yacyshyn et al., 1998; Reinisch et al., 2018). The remission rate after 1 month of therapy was 47% (7/15) in patients treated with the investigated substance and 20% (1/5) in patients treated with placebo. At the end of the 6 months follow up period, 5 of the 7 patients treated with the investigated substance maintained clinical remission and 33% (5 of 15) successfully tapered concomitant corticosteroid treatment (Yacyshyn et al., 1998).

These positive perspectives about the steroid sparing effect of ISIS 2302 were not confirmed in a subsequent trial, which primary endpoint was steroid-free remission at week 14. Here, 75 steroid refractory CD patients were randomized to four groups of treatment with ISIS 2302 or to placebo. The study drug was administered subcutaneously at four different dose-interval regimens (0.5 mg/kg once daily for 2 days; 0.5 mg/kg five times a week for 1 week; 0.5 mg/kg five times a week for 2 weeks or 0.5 mg/kg five times a week for 4 weeks) (Schreiber et al., 2001; Lobaton et al., 2014). Steroid-free remission at week 14 was registered in only 3.3% (2/60) of patients treated with ISIS 2302 and in none of the placebo patients (Schreiber et al., 2001; Lobaton et al., 2014). This negative property to induce steroid-free remission was also confirmed by another study (Yacyshyn et al., 2002b). Here, 299 steroid-dependent CD patients were randomized into three treatment arms: ISIS 2302 (2 mg/kg) given intravenously three times a week for two weeks, vs. ISIS 2302 (2 mg/kg) given intravenously three times a week for 4 weeks, compared to placebo application. Steroid free remission at week 14 was defined as the primary endpoint. It was reached by 20.2 and 21.2%, respectively in the ISIS 2302 arm with treatment lasting 2 or 4 weeks compared to 18.8% in the placebo arm. Therefore, no statistical significant difference regarding clinical efficacy could be shown (Yacyshyn et al., 2002b).

The last clinical trial conducted in CD patients, which results were published in 2007, confirmed the inefficacy of alicaforsen in inducing clinical remission in CD patients. In this study, 331 CD patients were randomized to two treatment groups: alicaforsen given intravenously, at a dosage of 100 mg at the time of the first infusion, followed by 300 mg given three times a week for 4 weeks (*n* = 221) compared to placebo administration (*n* = 110). The primary endpoint was clinical remission at week 12. No statistical differences regarding clinical remission at week 12 were evidenced between the two treatment groups (33.9% in the group treated with alicaforsen vs. 34.5% in the placebo group; *p* = 0.89) (Yacyshyn et al., 2007). These results have led to the halt of further clinical studies of this compound in CD patients.

In UC, some clinical studies demonstrated efficacy of alicaforsen in inducing clinical response and remission via topical application. First, an effective induction of clinical response by topical application of alicaforsen was evidenced by a randomized multicenter trial conducted in 40 UC patients affected by mild to moderate distal colitis, who were randomized to four dosing cohorts of an alicaforsen enema (0.1, 0.5, 2, or 4 mg/ml) or placebo, given once daily for 28 consecutive days (van Deventer et al., 2004). This therapeutic procedure resulted in the induction of clinical response in a dose-dependent way, with induction of response in 70% of alicaforsen 4 mg/ml treated patients compared to a placebo response of 28% at week 4, which was statistically significant (*p* = 0.004). In the group treated with alicaforsen at a dosage of 2 mg/ml, clinical response was evidenced in 45% of treated patients (*p* = 0.201). During the 6 months clinical follow up period, half of the patients in the placebo arm (4/8) required another medication or surgical intervention, whereas none of the patients treated with the highest dose of alicaforsen and two patients in the 2 mg/ml group required treatment escalation (van Deventer et al., 2004).

A randomized controlled trial conducted in active UC patients affected by mild to moderate left-sided colitis did not lead to a significantly different clinical outcome between the groups treated with topical application of the alicaforsen enema compared to placebo administration. The patients were randomized to five treatment arms: alicaforsen enema at a dosage of 120 mg daily for the first 10 days of 6 weeks of treatment and then every other day thereafter; 240 mg every other day for 6 weeks; 240 mg daily for the first 10 days of 6 weeks of treatment and then every other day thereafter or 240 mg daily for 6 weeks or placebo application. Primary endpoint was the Disease Activity Index (DAI) score at week 6. No significant differences were evidenced between the treatment arms and placebo (van Deventer et al., 2006). All groups demonstrated a decrease in the DAI score, but the best cohort response was identified in the group treated with daily application of 240 mg alicaforsen enema. Clinical response at week 6 was 47% in the 240 mg alicaforsen arm and 33% in the placebo arm (*p* = N.S.). Despite no significant differences identified for the primary endpoint clinical response, this study demonstrated that patients treated with an alicaforsen enema maintained an improved DAI score from week 18 to week 30 after commencement of therapy, compared to patients treated with placebo. The decrease in the mean DAI score compared to baseline was 51% at week 18 and 50% at week 30 in the group treated with daily application of 240 mg alicaforsen enema, compared to 18% (*p* = 0.04) and 11% in the placebo arm (*p* = 0.03) (van Deventer et al., 2006).

A subsequent randomized study was conducted in UC patients affected by mild to moderate distal UC, which were randomized to three treatment arms: 120 or 240 mg alicaforsen or 4 g mesalazine enema, given once daily for a total of 6 weeks (Miner et al., 2006). The aim of the study was to compare the effects of alicaforsen enema administration to standard mesalazine enema therapy. The primary endpoint was DAI reduction at week 6. No significant differences were highlighted between the treatment arms. The mean percentage reduction of the DAI score from baseline was 50% for the group treated with the mesalazine enema and 40% and 41% for the group treated with 120 and 240 mg of the alicaforsen enema (*p* = 0.27 and 0.32, respectively). Despite the failed difference regarding the primary endpoint, in the therapeutic arms of the alicaforsen enema the maintenance of clinical response was longer (128 and 146 days, respectively) in comparison to the mesalazine arm (54 days) (Miner et al., 2006). Moreover, the highest rate of mucosal healing in the 240 mg alicaforsen group was evidenced at week 10 and was achieved by 24% of patients; in the mesalazine arm it was achieved by 17% of patients and was evidenced at week 3 and 6 (*p* = 0.06) (Miner et al., 2006). These two studies indicated a possible modest durable effect of alicaforsen enema application and sustained maintenance of clinical response, but nevertheless non-significance regarding the primary endpoint.

A *post-hoc* meta-analysis focused on the results of four phase 2 clinical studies and investigated the overall efficacy of alicaforsen enema administration in the treatment of patients with active left-sided UC and pancolitis. The authors considered for the meta-analysis three different subgroups of patients: the subgroup with distal UC with active inflammation and disease extension from 5–40 cm; the subgroup with moderate or severe disease regardless of the extension of disease and the subgroup with both distal colitis and moderate/severe inflammation. The authors considered for the analysis the following treatment patterns: 240 mg alicaforsen enema once daily for a duration of 6 weeks; 4 g mesalazine enema once daily for 6 weeks or placebo. The higher efficacy (measured as mean percentage reduction in the DAI score) of alicaforsen over placebo was statistically significant at week 6 (51.4 vs. 27.1%; *p* = 0.004) and week 10 (51 vs. 24.7%; *p* = 0.014) in patients with distal disease. At week 30, alicaforsen enema application did not demonstrate a significant effect of maintenance of clinical response in comparison to placebo therapy (Vegter et al., 2013).

In the meta-analysis, a similar clinical efficacy (measured as mean percentage reduction in the DAI score) at week 6 and 10 was evidenced between alicaforsen and mesalazine enema therapy (51.4 vs. 51.3% at week 6 and 51 vs. 41.6% at week 10). However, the efficacy of alicaforsen enema was more durable than that of mesalazine, after 6 weeks of treatment. At week 30 the efficacy (mean percentage reduction in the DAI score) of alicaforsen enema application resulted to be 40.1%, while the efficacy of mesalazine enema was 20.6% (*p* = 0.05) in the treated patients. Similar results as for the subgroup with distal UC were found for the remaining two other subgroups of patients (Vegter et al., 2013).

Despite the profound improvement in UC management with biological drugs, a substantial proportion of patients require surgical treatment with proctocolectomy and ileal-pouch-anal anastomosis (IPAA) in the course of disease, in particular for severe flares of active disease, refractory to available medical therapies or due to the diagnosis of non-resectable colonic dysplasia. Onset of acute or chronic pouchitis is one of the most frequently occurring post-operative complications that patients experience and almost half of them present with this kind of problem within 5 years after the time of surgery (Tiainen and Matikainen, 1999). Alicaforsen could represent a novel therapeutic option for this hard to treat clinical problem, as indicated by conducted studies.

A first open-label uncontrolled study conducted in 12 patients affected by chronic pouchitis figured out a statistical significant reduction of the Pouch-Disease-Activity-Index (PDAI), which was 11.42 at baseline and 6.83 at week 6 (*p* = 0.001) and also of the PDAI endoscopic sub-score at week 6, which was 3.75 at baseline and 2.25 at week 6 (*p* = 0.0117) after daily topical administration of alicaforsen at a dosage of 240 mg for 6 weeks (Miner et al., 2004; McConnell et al., 2009). A phase III randomized trial conducted in patients affected by refractory pouchitis has been recently completed (Fiorino et al., 2011; EPG Health, 2018).

The main findings regarding the above mentioned clinical trials can be found in Table 1.

**Table 1:** Overview of clinical studies with alicaforsen.

**Study drug**	**Study**	**Phase**	**Study population**	**Study design**	**Cohort size**	**Drug dosage and frequency of doses**	**Primary endpoint**	**Results**
ISIS 2302	Phase I safety and pharmacokinetic profile of an intercellular adhesion molecule-1 antisense oligodeoxynucleotide (ISIS 2302) (Glover et al., 1997)	Phase 1	Healthy male volunteers	Double-blind, placebo-controlled study	44 male volunteers	Four healthy male volunteers recruited to each of seven single-dose groups (0.06, 0.12, 0.24, 0.5, 1.0, 1.5, and 2.0 mg/kg) and each of four multiple-dose groups (0.2, 0.5, 1.0, and 2.0 mg/kg). One subject in each group was allocated to placebo arm. The drug was infused intravenously on day 1 in the single dose-group and on days 1, 3, 5, and 7 in the multiple-dose group	Safety and pharmacokinetic profile of the drug	Good tolerability to the drug, with reproducibility of plasma pharmacokinetics
ISIS 2302	Dose ranging pharmacokinetic trial of high-dose alicaforsen (intercellular adhesion molecule-1 antisense oligodeoxynucleotide) (ISIS 2302) in active Crohn's disease (Yacyshyn et al., 2002a)	/	Patients with active CD (CDAI ≥220)	Double-blind, randomized study	22 CD patients	10 patients were randomized to the 300 mg arm, 10 to the 350 mg arm and 2 patients to the 250 mg arm. The drug was infused intravenously three times a week for 4 weeks	Clinical remission (CDAI ≤ 150) at week 8, 12, and after 6 months	In the intention to treat (ITT) population 14% (3/22) of the patients at week 8 and 23% (5/22) of the patients at week 12 were in clinical remission. After 6 months, 18% (4/22) of the ITT patients were in clinical remission. The efficacy was equivalent with all the three doses used
ISIS 2302	A placebo-controlled trial of ICAM-1 antisense oligonucleotide in the treatment of Crohn's disease (Yacyshyn et al., 1998)	/	Patients with active moderate CD (CDAI 220–350)	Double-blind, placebo-controlled study	20 CD patients	The patients were randomized (3:1, ISIS 2302 to placebo). The drug was infused intravenously at a dosage of 0.5, 1, or 2 mg/kg and was administered in 13 doses over 26 days	Assessment of tolerability, pharmacology and efficacy of the drug in steroid-dependent CD	Clinical remission was figured out in 47% (7/15) of the patients treated with the drug and in 20% (1/5) of those treated with placebo after 26 days from the start of the therapy. After 6 months 5 of the 7 patients treated with the drug maintained remission. The drug was correlated to a good profile of safety
ISIS 2302	Efficacy of subcutaneous antisense ICAM-1 treatment of chronic active Crohn's disease (Schreiber et al., 2001)	/	Patients with steroid-refractory CD (CDAI 200–400)	Double-blind, placebo-controlled study	75 CD patients	75 patients were randomized to 4 treatment arms and a placebo group. 14 patients received treatment for 2 days, 17 patients for 1 week, 15 patients for 2 weeks and 14 patients for 4 weeks. 15 patients received placebo. The drug was administered subcutaneously at a dosage of 0.5 mg/kg	Complete clinical remission (CDAI < 150 with discontinuation of steroids) at week 14	Clinical remission was reached by 18.3% of treated patients and by 20% of those treated with placebo at the week 14. No difference in clinical remission between the drug and placebo was evidenced
ISIS 2302	Double blind, placebo controlled trial of the remission inducing and steroid sparing properties of an ICAM-1 antisense oligodeoxynucleotide, alicaforsen (ISIS 2302), in active steroid dependent Crohn's disease (Yacyshyn et al., 2002b)	/	Steroid dependent CD patients with active disease (CDAI 200–350)	Double-blind, placebo-controlled study	299 CD patients	The patients were randomized into three groups: ISIS 2302 at a dosage of 2 mg/kg intravenously three times a week for 2 weeks, ISIS 2302 at the same dosage and with the same frequency but for 4 weeks and placebo	Steroid free remission with a CDAI < 150 at the end of week 14	Steroid free remission at week 14 was 20.2% in the group treated for 2 weeks, 21.2% in the group treated for 4 weeks and 18.8% in placebo group. No difference in clinical remission between the drug and placebo was evidenced
ISIS 2302 (Alicaforsen)	A randomized, double-masked, placebo-controlled study of alicaforsen, an antisense inhibitor of intercellular adhesion molecule 1, for the treatment of subjects with active Crohn's disease (Yacyshyn et al., 2007)	Phase 3	Patients with active CD (CDAI 220–400)	Double-blind, placebo-controlled study	331 CD patients	221 patients were randomized to the study drug at a dosage of 100 mg at the first infusion and for the other 11 infusions at a dosage of 300 mg (patients weighing more than 50 kg) or 200 mg (patients weighing 36–50 kg). The drug was administered intravenously 3 times a week for 4 weeks. 110 patients were enrolled in placebo arm	Clinical remission at week 12	No statistical difference between the study drug and placebo for clinical remission at week 12 (33.9% in the drug and 34.5% in the placebo arm)
ISIS 2302 (Alicaforsen)	A randomized, controlled, double blind, escalating dose study of alicaforsen enema in active ulcerative colitis (van Deventer et al., 2004)	/	Patients with active distal UC (DAI 4–10)	Double-blind, placebo-controlled study	40 UC patients	The patients were randomized to the alicaforsen enema administration at four different dosages (0.1, 0.5, 2, or 4 mg/ml) or to placebo. Each patient received alicaforsen enema once daily for 28 days	Safety and efficacy of the drug enema after 1, 3, and 6 months	At 1 month alicaforsen enema (4 mg/ml) was correlated with 70% of improved disease activity index compared to 28% with placebo, with statistical significant difference. This positive outcome was evidenced also after 3 and 6 months
ISIS 2302 (Alicaforsen)	A Phase II dose ranging, double-blind, placebo-controlled study of alicaforsen enema in subjects with acute exacerbation of mild to moderate left-sided ulcerative colitis (van Deventer et al., 2006)	Phase 2	Patients with active distal UC (DAI 4–10) with disease flare	Double-blind, placebo-controlled study	120 UC patients	The patients were randomized to five treatment arms: placebo, alicaforsen enema at a dosage of 120 mg daily for 10 days and then every other day, 240 mg every other day, 240 mg daily for 10 days and then every other day, 240 mg daily	Disease Activity Index at week 6	No significant difference between alicaforsen and placebo enema was evidenced at week 6, but the arm of alicaforsen (240 mg with daily administration) evidenced a statistical benefit over placebo for prolonged reduction of DAI from the week 18 to week 30
ISIS 2302 (Alicaforsen)	Safety and efficacy of two dose formulations of alicaforsen enema compared with mesalazine enema for treatment of mild to moderate left-sided ulcerative colitis: a randomized, double-blind, active-controlled trial (Miner et al., 2006)	/	Patients with active UC (DAI 4–10)	Double-blind, active-controlled study	159 UC patients	The patients were randomized to alicaforsen enema at a dosage of 120 mg or 240 mg or to mesalazine enema at a dosage of 4 g. The enema was administered daily for 6 weeks	DAI at week 6 relative to baseline	No difference in reducing clinical activity was evidenced between the treatment strategies at week 6, but alicaforsen enema evidenced more prolonged response than mesalazine enema
ISIS 2302 (Alicaforsen)	An enema formulation of alicaforsen, an antisense inhibitor of intercellular adhesion molecule-1, in the treatment of chronic, unremitting pouchitis (Miner et al., 2004)	/	Patients with chronic, unremitting pouchitis with a Pouchitis Disease Activity Index (PDAI) score of ≥7	Open-label, uncontrolled study	12 patients affected by chronic unremitting pouchitis	The patients received daily administration of alicaforsen enema at a dosage of 240 mg for 6 weeks	Efficacy of alicaforsen enema in the treatment of chronic unremitting pouchitis	A significant benefit of alicaforsen enema was evidenced at week 6. The PDAI score resulted to be 6.83 at week 6 compared to 11.42 at baseline. This benefit was evidenced also for improvement of endoscopic activity

*CD, Crohn's disease; UC, ulcerative colitis; CDAI, Crohn's disease Activity Index; DAI, Disease Activity Index; PDAI, Pouchitis Disease Activity Index; ICAM1, Intercellular Cell Adhesion Molecule-1; /, phase indication not found*.

## Mongersen

Immune homeostasis in the intestinal mucosa plays a fundamental role in preventing inflammatory immune responses in IBD (MacDonald and Monteleone, 2005; MacDonald et al., 2011). One of the most important cytokines that mediate a regulatory mucosal immune response is Transforming Growth Factor β (TGFβ). This cytokine has been implicated in the function of regulatory T (T reg) cells that mediate suppression of inflammatory responses, as evidenced in mice models of colitis (Powrie et al., 1996). Different studies figured out a greater level of regulatory T cells in the inflamed mucosa of IBD patients than in healthy controls, thereby inducing subsequent studies for the analysis of T reg cells in the tissue of IBD patients (Monteleone et al., 2001).

A study conducted in mice models of colitis demonstrated increased TGFβ levels in the inflamed tissue of colitic mice, indicating that a blockade of TGFβ signaling transduction by counter-regulatory proteins overexpressed during the active state of inflammation might be involved to suppress its anti-inflammatory function (Boirivant et al., 2006). This finding has been the first step toward the discovery of Mothers against decapentaplegic homolog 7 (Smad7) that has an inhibitory role within the TGFβ signaling cascade. This intracellular signaling molecule is an inhibitor of TGFβ signaling, as it can bind to the TGFβ receptor thereby inhibiting phosphorylation of downstream signaling molecules such as Smad2 and Smad3, whose activation mediates TGFβ signal transduction.

First studies figured out a heightened expression of Smad7 in the mucosal intestinal compartment of IBD patients (Monteleone et al., 2001). In contrast, high expression of phosphorylated Smad3 was seen in the normal intestinal mucosa (Monteleone et al., 2001). The same study showed an increased response of intestinal mononuclear cells to TGFβ after the administration of a Smad7 ASO, which restored the phosphorylation of Smad3. These findings demonstrate that Smad7 blockade by a Smad7 ASO enabled TGFβ to inhibit the function of pro-inflammatory intestinal cells.

A subsequent phase I open-label study of an ASO against Smad7 (GED0301) was conducted in CD patients with steroid-dependent/resistant disease. Fifteen CD patients were randomized to receive one of the three treatment dosages of oral GED0301 (40, 80, or 160 mg) once daily for 7 consecutive days. The drug was well-tolerated. Oral administration of GED0301 (mongersen) led to the reduction of pro-inflammatory C-C Chemokine Receptor 9 (CCR9)-positive T cells in the bloodstream. This study showed a good safety and tolerability profile of the studied substance (Monteleone et al., 2012). Since TGFβ has been described to induce the profibrogenetic response of fibroblasts, patients were monitored for 6 months with intestinal ultrasonography, not showing signs of intestinal stenosis. Moreover, half of the patients maintained remission during the 6 months follow up period (Zorzi et al., 2012).

Afterwards, a randomized, double-blind, phase 2 study was conducted in CD patients with inflammatory lesions in the terminal ileum and/or right colon, with steroid-dependence/resistance, randomized to three different daily oral drug doses (10, 40, or 160 mg of mongersen or placebo) for 2 weeks. The primary outcome was clinical remission at day 15, with maintenance of remission for at least 2 weeks. Clinical remission was not significantly different between the patients in the 10 mg mongersen group and placebo (12 vs. 10%; *p* = N.S.); however clinical remission was reached by 55% and 65% of patients in the 40 and 160 mg mongersen group, as compared to 10% in the placebo group (*p* < 0.001). The drug showed a good safety and tolerability profile (Monteleone et al., 2015).

A following study demonstrated efficacy of GED0301 in inducing both clinical and endoscopic response in a study group of 63 CD patients. The patients were randomized to three treatment groups (4, 8, or 12 weeks of treatment with oral GED-0301 at a dose of 160 mg/day). 37% of patients had an endoscopic response, defined as ≥25% reduction in Simple Endoscopic Score for Crohn's disease (SES-CD), at week 12, with a substantial proportion of patients in clinical remission at the same time point (32% in the group of 4 weeks of treatment, 35 and 48%, respectively in the group of 8 and 12 weeks of treatment) (Feagan et al., 2018). This study however lacked a placebo control group.

Despite these promising results, a recently conducted phase 3 study was stopped following the finding of non-effectiveness of the studied drug. No safety findings were reported (Bevivino et al., 2018; White et al., 2018).

The main findings regarding the above mentioned clinical trials can be found in Table 2.

**Table 2:** Overview of clinical studies with mongersen.

**Study drug**	**Study**	**Phase**	**Study population**	**Study design**	**Cohort size**	**Drug dosage and frequency of doses**	**Primary endpoint**	**Results**
GED0301	Phase I clinical trial of Smad7 knockdown using antisense oligonucleotide in patients with active Crohn's disease (Monteleone et al., 2012)	Phase 1	CD patients with moderate to severe active disease (CDAI>220)	open-label study	15 CD patients	The patients were randomized to three treatment groups: GED0301 at a dosage of 40, 80, or 160 mg. The drug was administered orally once daily for 7 days	Safety and tolerability of the drug	The study evidenced good tolerability and safety of the drug
GED0301	A phase 1 open-label trial shows that smad7 antisense oligonucleotide (GED0301) does not increase the risk of small bowel strictures in Crohn's disease (Zorzi et al., 2012)	Phase 1	CD patients with an inflammatory phenotype and dependence and/or resistance to steroids	open-label study	15 CD patients	The patients were randomized to three treatment groups: GED0301 at a dosage of 40, 80, or 160 mg. The drug was administered orally once daily for 7 days	To evaluate whether GED0301 is associated with the formation of small bowel strictures	The treatment with GED0301 is not associated with the development of small bowel stricture as assessed by intestinal sonography
Mongersen (GED0301)	Mongersen, an oral SMAD7 antisense oligonucleotide, and Crohn's disease (Monteleone et al., 2015)	Phase 2	CD patients with ileitis and/or right-sided colonic disease and moderate to severe active disease (CDAI 220–400)	Double-blind, placebo-controlled study	166 CD patients	The patients were randomized to four treatment arms: mongersen at a dosage of 10 mg per day, 40 mg per day, 160 mg per day or placebo. The drug was administered orally once daily for 2 weeks	Clinical remission (CDAI < 150) at day 15 and maintenance of remission for at least 2 weeks	Clinical remission at day 15 and maintenance for 2 weeks was evidenced in 55% of the patients treated with 40 mg of the drug, 65% of those treated with 160 mg and in 10% of placebo patients. This difference was statistically significant. Clinical remission at days 28 and 84 was significantly higher in the patients treated with 40 or 160 mg of the drug than in the group treated with 10 mg of the drug or with placebo
Mongersen (GED0301)	Effects of mongersen (GED-0301) on endoscopic and clinical outcomes in patients with active Crohn's disease (Feagan et al., 2018)	Phase 1	CD patients with active disease (CDAI≥220 to ≤ 450)	Double-blind study	63 CD patients	The patients were randomized to three treatment groups: GED0301 at a dosage of 160 mg per day for 4 weeks, 160 mg per day for 8 weeks and 160 mg per day for 12 weeks. The drug was administered orally	To evaluate endoscopic outcomes at week 12	The study evidenced endoscopic improvement (25% reduction SES-CD) in 37% of the patients with evaluable endoscopy at week 12. No significant difference was evidenced between treatment groups

*CDAI, Crohn's disease Activity Index; Smad7, Mothers against decapentaplegic homolog 7; CD, Crohn's disease*.

## NFkB ASO

Preclinical studies, conducted in murine models of colitis, demonstrated the efficacy of an ASO against the p65 subunit of the transcription factor Nuclear Factor Kappa B (NFkB) (Neurath et al., 1996; Murano et al., 2000). This transcription factor mediates a key role in the pro-inflammatory response of immune-mediated disorders (Zhang et al., 2017).

A preclinical study evidenced an upregulation of p65 expression in macrophages located in the Lamina propria of patients affected by CD. The authors demonstrated the efficacy of a human p65 ASO to reduce the production of pro-inflammatory cytokines by these cells (Neurath et al., 1996). Another study conducted in a murine model of colitis demonstrated a down-regulation of chronic inflammation- induced intestinal fibrosis by the p65 ASO (Lawrance et al., 2003).

Despite these promising results, no clinical study has been published so far regarding the use of this therapeutic strategy in IBD patients (Marafini and Monteleone, 2018).

## Cobitolimod

Cobitolimod (formerly referred to as DIMS0150) is a single-stranded oligonucleotide that is recognized by the Toll-like Receptor 9 (TLR9) and represents a possible promising novel therapy for UC patients. The altered response of intestinal immune cells to the commensal bacterial flora has been considered to be one of the pivotal pathogenic mechanisms in UC (Strober et al., 2007). The protective immune cell response plays the counterpart action against the over activation of the mucosal immune system (Neurath, 2014). Toll-like receptors (TLRs) belong to pattern recognition receptors (PRRs), which mediate the recognition process of self-antigens from foreign-antigens by the host. These receptors can recognize the pathogen associated molecular patterns (PAMPs) and are implicated in the first defensive innate immune response against luminal pathogens, but can also induce mechanisms of cellular and mucosal repair (Lee et al., 2006). TLR9 is a member of the TLR family and recognizes bacterial DNA, but also viral DNA (e.g., Cytomegalovirus DNA). This receptor in particular recognizes bacterial Cytosine Guanine (CpG) dinucleotides motifs (Hemmi et al., 2000).

A study in the murine model of Salmonella typhimurium infection figured out that the activation of TLR9 maintained the homeostasis of intestinal mucosal cells, demonstrating that disruption of TLR9 signaling could result in augmented inflammation and worsening of intestinal injury (Li et al., 2017).

Cobitolimod is a 19 base single-strand oligonucleotide which mimics bacterial DNA as its CpG dinucleotide sequences are recognized by the TLR9 expressed by different immune cells, thereby mediating anti-inflammatory effects. In preclinical studies in murine models of colitis, administration of the TLR9 oligonucleotide agonist suppressed the severity of colitis in RAG-/- mice. The type I Interferon (IFN α/β) was induced by TLR9 activation and mediated anti-inflammatory responses (Katakura et al., 2005). In another preclinical study conducted in the experimental DSS model and spontaneous colitis mice, administration of CpG oligonucleotides improved clinical, biochemical and histologic activity of colonic inflammation (Rachmilewitz et al., 2002).

A study published in 2013 considered the possible efficacy of DIMS0150 in treating UC patients who had been considered as treatment failures with indication for colectomy. Seven patients received a single dose of topical DIMS0150 (30 mg) and one patient three doses with an interval of 4 weeks in between. The study drug was applied during colonoscopy with a spray catheter in the upper descending colon or the transverse colon. Efficacy was recorded as an improvement of the DAI score and of endoscopic and histological activity at week 12. Clinical response was found in all patients 1 week after single endoscopic administration of the drug. After 12 weeks, 82% of patients had a clinical response while 71% were in clinical remission. In the 2-year follow up one patient needed colectomy (Musch et al., 2013).

A non-interventional pilot study analyzed the transcripts of 34 steroid response genes by polymerase chain reaction (PCR) in the blood derived peripheral mononuclear cells (PBMCs) of steroid refractory UC patients, after their incubation with DIMS0150 (25 or 100 μM) in the presence of Dexamethasone. The authors evidenced that addition of DIMS0150 to steroid treatment produced a significant enhancement of mRNA levels of three steroid response genes (CD163, TSP-1, and IL-1RII) and suggested to consider these genes as potential biomarkers of response to the study drug. This study highlighted the potential effect of DIMS0150 to restore steroid sensitivity in steroid refractory UC patients (Kuznetsov et al., 2014).

After these findings a randomized, double-blind, placebo-controlled trial (COLLECT study) was conducted in 131 patients affected by moderate to severe UC. Patients were randomized to receive two topical endoscopic administrations of cobitolimod (DIMS0150) at a dosage of 30 mg at baseline and week 4 or respective placebo application. The administration was done proximally to the site of mucosal inflammation, or in the transverse colon in the event of pancolitis, using a spray catheter during endoscopy (Atreya et al., 2016). There was no statistical difference in the primary endpoint clinical remission, defined as a CAI score of ≤ 4 at week 12, which was recorded in 44.4% of cobitolimod treated patients and in 46.5% of those treated with placebo (Atreya et al., 2016). However, symptomatic remission, which was defined as the absence of blood in stool and a weekly frequency of < 35 stools, was shown in a larger proportion of patients treated with the drug compared to those treated with placebo (32.1% in the cobitolimod group vs. 14% in the placebo group at week 4 (*p* = 0.02) and 44.4 vs. 27.9%, respectively at week 8 (*p* = 0.06). Mucosal healing at week 4 was also achieved by more cobitolimod treated patients compared to the placebo group, but missed statistical significance (34.6 vs. 18.6%; *p* = 0.09). Histological improvement, defined as a Geboes score of 0–2, was evident in 30.9% of the cobitolimod treated vs. 9.3% of the placebo treated patients (*p* = 0.007). The combination of clinical and endoscopic remission could be observed in 21% of cobitolimod treated patients vs. 4.7% of patients on placebo (*p* = 0.021) at week 4 (Atreya et al., 2016).

A recent *post-hoc* analysis, based on data from the COLLECT study, has shown that the clinical efficacy of the drug is significant if patient-reported-outcomes (PRO) were considered, as the investigated outcome measure. Symptomatic remission was studied in 104 patients based on their e-diary records and was defined as the absence of blood in stool and a mean daily stool frequency < 4 (Atreya et al., 2018). Symptomatic remission was found at week 4 in 17.1% of patients treated with cobitolimod compared to 5.9% of patients treated with placebo (*p* = 0.13) and, respectively, in 35.7 and 17.6% (*p* = 0.07) at week 8 and in 38.6 and 17.6% (*p* = 0.04) at week 12 (Atreya et al., 2018).

BL7040 (Monarsen) is an orally applied oligonucleotide that modulates the TLR9 and it was investigated in a prospective multicenter phase 2 study in patients with moderately active UC at doses of 12 mg once daily for 3 weeks followed by 40 mg once daily for 2 weeks. The primary endpoint was the proportion of patients with clinical response at the end of 5 weeks of treatment. Clinical response was defined by a decrease of at least three points and 30 % reduction of the Mayo score compared to baseline. Half of the patients (8/16) achieved clinical response at the end of the course of treatment, with a good tolerability profile of the drug (Dotan et al., 2016).

Altogether, agonists of the Toll-like receptor-9 appeared safe and well-tolerated in moderate to severe UC patients and could represent a novel promising therapeutic option for the management of UC patients.

Currently, a phase 2 study (CONDUCT) of different doses and different administration intervals of cobitolimod in an enema formulation is tested in patients affected by moderate to severe UC^1^.

The main findings regarding the above mentioned clinical trials can be found in Table 3.

**Table 3:** Overview of clinical studies with cobitolimod.

**Study drug**	**Study**	**Phase**	**Study population**	**Study design**	**Cohort size**	**Drug dosage and frequency of doses**	**Primary endpoint**	**Results**
DIMS0150	Topical treatment with the Toll-like receptor agonist DIMS0150 has potential for lasting relief of symptoms in patients with chronic active ulcerative colitis by restoring glucocorticoid sensitivity (Musch et al., 2013)	Phase 1	UC patients with chronic active disease, with indication for for colectomy	Open-label study	8 UC patients	7 patients were treated with single topical administration of 30 mg of DIMS0150 and one patient with three doses of 30 mg of DIMS0150 with an interval of 4 weeks between doses	Efficacy of the drug at week 12	82% of clinical response and 71% of clinical remission were evidenced at week 12. After two years all but one of treated patients avoided colectomy
DIMS0150 (cobitolimod)	Clinical effects of a topically applied Toll-like receptor 9 agonist in active moderate-to-severe Ulcerative Colitis (Atreya et al., 2016)	Phase 3, COLLECT study	UC patients with moderate to severe disease (CAI≥9 with an endoscopic Mayo score ≥2)	Double-blind, placebo-controlled study	131 UC patients	The patients were randomized to the treatment arm with DIMS0150 at a dosage of 30 mg or to placebo arm. The drug was administered topically during lower gastrointestinal endoscopy at baseline and at week 4	Clinical remission (CAI ≤ 4) at week 12	Clinical remission at week 12 was 44.4% in the group treated with DIMS0150 and 46.5% in the group treated with placebo. No significant difference in clinical remission was evidenced between the groups
DIMS0150 (cobitolimod)	Clinical efficacy of the Toll-like receptor 9 agonist cobitolimod using patient-reported-outcomes defined clinical endpoints in patients with ulcerative colitis (Atreya et al., 2018)	Post-hoc analysis of the COLLECT study (Atreya et al., 2016)	UC patients with moderate to severe disease (CAI≥9 with an endoscopic Mayo score ≥2) with available e-diary data at baseline	Retrospective analysis of the COLLECT study	104 UC patients	The patients were randomized to the treatment arm with DIMS0150 at a dosage of 30 mg or to placebo arm. The drug was administered topically during lower gastrointestinal endoscopy at baseline and at week 4	Post hoc analysis of Symptomatic remission (absence of blood in stool and a mean daily stool frequency < 4) at week 4, 8, and 12	Symptomatic remission at week 4 was evidenced in 17.1% of the patients treated with cobitolimod and in 5.9% of those treated with placebo (p=0.13). At week 8 it was 35.7% vs. 17.6% (p = 0.07). At week 12 it was 38.6% vs. 17.6% (p = 0.04).
BL7040	Ameliorating active ulcerative colitis via an orally available Toll-like receptor-9 modifier: A prospective open-label, multicenter phase II trial (Dotan et al., 2016)	Phase 2	UC patients with moderate disease (Mayo score ≥5 and ≤ 9, endoscopic sub-score ≥2)	Open-label study	22 UC patients	BL7040 was administered orally at a dosage of 12 mg per day for 19–21 days, followed by 40 mg of BL7040 per day for 14 days	To evaluate the efficacy and safety of BL7040 in patients affected by UC	The study evidenced efficacy and safety of the drug

*CAI, Clinical Activity Index; UC, ulcerative colitis*.

## GATA3 DNAzyme

The adaptive immune response and inappropriate production of pro-inflammatory cytokines have an important pathogenic role in IBD pathogenesis. CD is characterized by a prevalence of T helper 1 (Th1) cytokines such as Interferon gamma (IFNγ) and TNF, whereas UC is marked by a of more Th2 oriented cytokine profile, characterized by IL4, IL5, and IL13 (Fuss et al., 1996, 2004). However, these subsets of T cells are not the only responsible cells for the inflammatory response in IBD. Th17 cells are another lymphocyte subpopulation which has a major role in intestinal inflammation in both CD and UC (Giuffrida et al., 2018). A recent published review revealed also the important role of other lymphocyte subsets in mucosal inflammation (Giuffrida et al., 2018). Importantly, Th9 cells and IL9 have also been described to play a pivotal role in the pathogenesis of UC (Nalleweg et al., 2015; Weigmann and Neurath, 2017).

The GATA3 specific DNAzyme (SB010) is an oligonucleotide which can mediate the cleavage of the mRNA of the transcription factor GATA3 and its possible therapeutic usage in human diseases was first investigated in patients affected by allergic asthma. A double-blind placebo-controlled study randomized 40 patients, affected by allergic asthma, to receive 10 mg of SB010 or placebo. The drug was administered by inhalation once daily for 28 days. Treatment with SB010 was found to be effective in attenuating the late and early asthmatic responses to allergen provocation; thereby the drug induced also a significant reduction of IL5 blood levels (Krug et al., 2015).

The Th2 response is mediated by the transcription factor GATA3, which has proven to be over-expressed in the colonic mucosa of UC patients (Ho et al., 2009; Ohtani et al., 2010). This transcription factor is not only linked to Th2 cell responses, but also plays an important role in regulating the differentiation of Th9 lymphocytes (Wan, 2014).

A study conducted with intestinal biopsies of UC patients as well as murine models of colitis showed a correlation between the expression of the transcription factor GATA3 and the production of inflammatory Th2 and Th9 related cytokines (Popp et al., 2016). Conditional GATA3 deficiency in T cells prevented experimental colitis in mice. Intrarectal administration of a GATA3 specific DNAzyme (hgd40) significantly ameliorated colitis in oxazolone and 2,4,6-trinitrobenzene sulfonic acid (TNBS)-mediated colitis models (Popp et al., 2016). The authors also demonstrated that the inhibition of GATA3 transcription by hgd40 was able to ameliorate colitis also in TNF receptor (TNFR) double-knockout mice. This result evidenced the efficacy of the experimental drug in reducing colitis in mice, independently of the presence of TNF (Popp et al., 2016).

These results underline the important role of GATA3 in the inflammatory response in UC and suggest a possible future role of the GATA3 DNAzyme in the therapy of UC patients.

A study of this novel drug as an enema formulation (SB012, SECURE study) in patients with moderate to severe UC has recently been completed (NCT02129439)^2^.

## STNM01: an Oligonucleotide Against Carbohydrate Sulfotransferase 15

IBD pathogenesis is not only connected to an active acute and chronic inflammatory response but also to structural damage, with matrix remodeling and tissue fibrosis, which can take place independently of the inflammatory activity (Suzuki and Yoneyama, 2017). Approximately one third of patients with CD and 5% of those with UC are diagnosed with fibrotic stenosis in the clinical course (Rieder et al., 2013; de Bruyn et al., 2015). There are no specific therapies for the management of fibrotic intestinal remodeling, induced by the over activation of stromal cells during chronic inflammation. Invasive treatment (endoscopic dilation and/or surgical treatment) represent the inevitable final management step of this complication.

Carbohydrate sulfotransferase 15 (CHST15) is an intracellular enzyme that mediates the biosynthesis of sulfated matrix glycosaminoglycans (GAG) which can induce fibrotic reactions in IBD patients.

STNM01 is a novel double-strand RNA oligonucleotide that selectively blocks the expression of CHST15 mRNA and can inhibit the excessive production of glycosaminoglycans in the colon by fibroblasts (Suzuki et al., 2016).

For this reason an endoscopic approach of drug delivery through submucosal injection, to directly target the gut lesion, was established in mice in 2016 (Suzuki et al., 2016). CHST15 siRNA or negative control siRNA were endoscopically administered by submucosal injection at day 6 in DSS-mediated colitis models. The mice were sacrificed at day 19 to evaluate the effect of the experimental drug. CHST15 siRNA reduced CHST15 mRNA in the colon of treated mice and significantly ameliorated endoscopic and histologic disease activity (Suzuki et al., 2016).

A subsequent published randomized placebo-controlled trial evaluated the safety of STNM01 in CD patients whose mucosal ulcerative lesions were refractory to conventional therapy. Eighteen CD patients were randomized to receive a single endoscopic submucosal injection of STNM01 (2.5, 25, or 250 nM) or placebo. Each dose was administered to eight sites directly surrounding the biggest ulcerative lesion evidenced by total colonoscopy. STNM01 was able to reduce the segmental SES-CD score at day 30 when compared to the placebo arm and was also able to induce a reduced extension of fibrosis, documented by histological analyses. The experimental drug also showed a good safety profile (Suzuki et al., 2017).

A phase 2 clinical trial in UC patients affected by refractory left-sided UC has recently been completed. The patients were randomized to submucosal injections of STNM01 or placebo in the last 35 cm of the colon.

The main findings regarding the above mentioned clinical trials can be found in Table 4.

**Table 4:** Overview of clinical studies with STNM01.

**Study drug**	**Study**	**Phase**	**Study population**	**Study design**	**Cohort size**	**Drug dosage and frequency of doses**	**Primary endpoint**	**Results**
STNM01	Phase 1 clinical study of siRNA targeting carbohydrate sulfotransferase 15 in Crohn's disease patients with active mucosal lesions (Suzuki et al., 2017)	Phase 1	CD patients with active endoscopic disease (SES-CD: from 1 to 11)	Double-blind, placebo-controlled study	18 CD patients	The patients were randomized to four different treatment arms: STNM01 at a dosage of 2.5 nM, STNM01 at a dosage of 25 nM, STNM01 at a dosage of 250 nM or placebo arm. The drug was administered endoscopically with a single dose submucosal injection	To evaluate the safety of STNM01	The study evidenced the safety of the drug and reduced the segmental SES-CD score

*SES-CD, Simple Endoscopic Score for Crohn's disease; siRNA, silenceRNA; CD, Crohn's disease*.

## Conclusions

In this review, we focused on recent and past approaches to test the therapeutic efficacy of oligonucleotide based therapies in IBD.

The combining mechanistic mode of oligonucleotide based therapeutics is a targeted action on specific pro-inflammatory molecules, which are over activated in IBD patients and contribute significantly to disease pathogenesis. The proposed high selectivity of the agents is derived from its mode of action, that aims to specifically block certain inflammatory molecular patterns, without a general systemic effect on other molecular targets. It would be important for each oligonucleotide to present more data regarding its specific mode of molecular action. Ideally, interaction with the proposed molecular target should be presented. Overall, the good tolerance and safety profile has allowed increasing development of this therapeutic approach in IBD clinical research and trials.

This new treatment strategy has the potential to address the main concern of a safe long-term therapy for IBD patients. Moreover, efficacious therapy could be prolonged as lasting maintenance therapy, as there is no risk for secondary loss-of-response due to the formation of anti-drug antibodies, which is one of the main reasons for limited long-term efficacy of biological drugs (Atreya and Neurath, 2018).

The above-mentioned properties and selectivity of oligonucleotides could respond to the dire need for a safe and efficacious long-term therapy. The numerous clinical trials conducted so far have shown the possibility of oligonucleotide treatment to match the target of safety and efficacy, but till now no oligonucleotide based therapy has been approved for clinical practice in the treatment of IBD patients and we still await successful late-stage clinical studies that demonstrate their efficaciousness.

## Author Contributions

PS wrote the manuscript. HS, GM, and MN assessed the articles and their relevance to the above topics. RA supervised and drafted the manuscript and is the corresponding author.

### Conflict of Interest Statement

RA reports grants or personal fees from AbbVie, Biogen, Boehringer Ingelheim GmbH & Co. KG, Cellgene, DrFalk Pharma GmbH, Ferring GmbH, GlaxoSmithKline plc, InDex Pharmaceuticals AG, Janssen-Cilag GmbH, MSD Sharp & Dome GmbH, Philogen, Pfizer Inc., Roche Pharma, Samsung Bioepsis, Stelic Institute, Sterna Biologicals, Takeda Pharma GmbH & Co. KG, and Tillotts Pharma AG outside the submitted work. MN reports personal fees from MSD Sharp & Dohme GmbH, PPM Services S.A., Index Pharmaceuticals AB, Shire GmbH, Boehringer Ingelheim GmbH & Co. KG, Janssen Cilag GmbH, Pentax Europe GmbH, Tillotts Pharma AG, e.Bavarian Health GmbH, Takeda Pharma GmbH & Co. KG, and grants from German Research Council, German Cancer Aid, outside the submitted work. GM has served as an advisory board member for ABBVIE. The remaining authors declare that the research was conducted in the absence of any commercial or financial relationships that could be construed as a potential conflict of interest.

## Footnotes

^1^ClinicalTrials.gov Identifier: NCT03178669.

^2^https://www.clinicaltrials.gov/ct2/show/NCT02129439?term=NCT02129439&rank=1
