# Supplementary material for: Oligonucleotides—A Novel Promising Therapeutic Option for IBD
Source: Front Pharmacol. 2019 Apr 24;10:314. doi: 10.3389/fphar.2019.00314 (PMC6491809; doi:10.3389/fphar.2019.00314)
Supplement: Supplementary file 1 [file Data_Sheet_1.DOCX]

**Supplementary materials**

**Materials and methods**

A comprehensive literature search was conducted in Embase, Medline (service of the US National Library of Medicine and the National Institutes of Health). The following key words were used (all fields): (‘Crohn’s disease (CD)’ OR ‘Crohn’s’ OR ‘Ulcerative colitis’ OR ‘Inflammatory Bowel Disease (IBD)’) and (‘oligonucleotide’ OR ‘antisense oligonucleotide’ OR ‘novel target therapy’). RA, MFN, GM, HS and PS assessed the articles and their relevance to the above topic.
